# Clostridioides difficile Modifies its Aromatic Compound Metabolism in Response to Amidochelocardin-Induced Membrane Stress

**DOI:** 10.1128/msphere.00302-22

**Published:** 2022-08-22

**Authors:** Madita Brauer, Sven-Kevin Hotop, Martina Wurster, Jennifer Herrmann, Marcus Miethke, Rabea Schlüter, Silvia Dittmann, Daniela Zühlke, Mark Brönstrup, Michael Lalk, Rolf Müller, Susanne Sievers, Jörg Bernhardt, Katharina Riedel

**Affiliations:** a Institute of Microbiology, University of Greifswald, Greifswald, Germany; b Institute of Marine Biotechnology, Greifswald, Germany; c Helmholtz Centre for Infection Research, Department of Chemical Biology, Braunschweig, Germany; d Institute of Biochemistry, University of Greifswald, Greifswald, Germany; e Helmholtz Institute for Pharmaceutical Research Saarland (HIPS) - Helmholtz Centre for Infection Research (HZI) and Department of Pharmacy, Saarland University, Saarbrücken, Germany; f German Center for Infection Research (DZIF), Hannover-Braunschweig, Germany; g Imaging Center of the Department of Biology, University of Greifswald, Greifswald, Germany; University of Iowa

**Keywords:** *Clostridioides difficile*, chelocardin, amidochelocardin, proton motive force, aromatic amino acids, phenazine, ClnRAB, membrane potential, proteomics

## Abstract

Amidochelocardin is a broad-spectrum antibiotic with activity against many Gram-positive and Gram-negative bacteria. According to recent data, the antibiotic effect of this atypical tetracycline is directed against the cytoplasmic membrane, which is associated with the dissipation of the membrane potential. Here, we investigated the effect of amidochelocardin on the proteome of Clostridioides difficile to gain insight into the membrane stress physiology of this important anaerobic pathogen. For the first time, the membrane-directed action of amidochelocardin was confirmed in an anaerobic pathogen. More importantly, our results revealed that aromatic compounds potentially play an important role in C. difficile upon dissipation of its membrane potential. More precisely, a simultaneously increased production of enzymes required for the synthesis of chorismate and two putative phenazine biosynthesis proteins point to the production of a hitherto unknown compound in response to membrane depolarization. Finally, increased levels of the ClnAB efflux system and its transcriptional regulator ClnR were found, which were previously found in response to cationic antimicrobial peptides like LL-37. Therefore, our data provide a starting point for a more detailed understanding of C. difficile*’s* way to counteract membrane-active compounds.

**IMPORTANCE** C. difficile is an important anaerobe pathogen causing mild to severe infections of the gastrointestinal tract. To avoid relapse of the infection following antibiotic therapy, antibiotics are needed that efficiently eradicate C. difficile from the intestinal tract. Since C. difficile was shown to be substantially sensitive to membrane-active antibiotics, it has been proposed that membrane-active antibiotics might be promising for the therapy of C. difficile infections. Therefore, we studied the response of C. difficile to amidochelocardin, a membrane-active antibiotic dissipating the membrane potential. Interestingly, C. difficile*’s* response to amidochelocardin indicates a role of aromatic metabolites in mediating stress caused by dissipation of the membrane potential.

## INTRODUCTION

**FIG 1:**
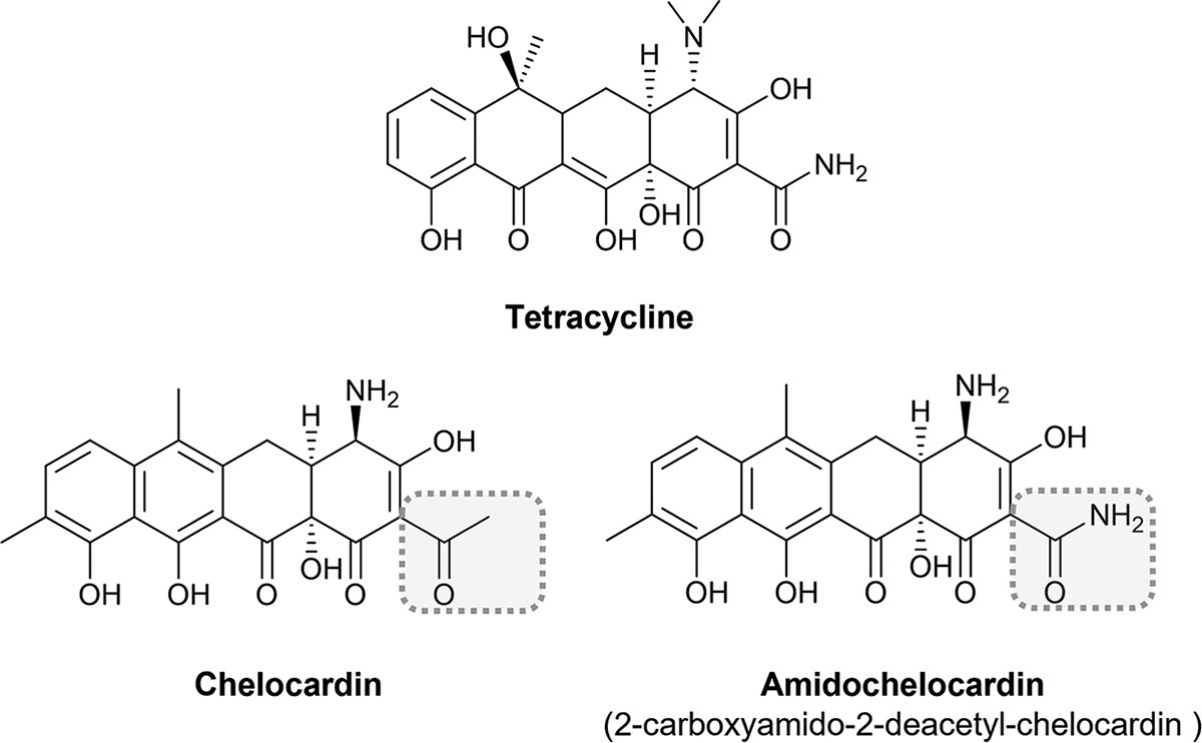
Chemical structures of tetracycline and the atypical tetracycline chelocardin and its amidated derivative amidochelocardin.

Antibiotic resistance has become an important health threat. New antibiotics, which overcome existing antibiotic resistance and are less prone to select for new resistance markers, are urgently required (1, 2). In this context, a few target sites within the cell are especially promising due to their essential role in cell metabolism and/or the expected low potential of antibiotic resistance development (1). For instance, antibiotics targeting structures of the cell envelope, especially the cell membrane, are of great interest (3, 4). In addition to its role as outer barrier, the cell membrane is a highly organized cellular structure with numerous functions in energy production, cell trafficking and signaling (5, 6). The bacterial cell membrane is indeed one of the oldest frontlines in the fight between pathogens and the host. Antimicrobial peptides produced by the innate immune system form the first line of defense against numerous pathogens. This large and diverse group of host-derived antimicrobials successfully disturbs the bacterial membrane function via pore formation, depolarization or effects on its fluidity (7). Likewise, several antibiotics can disturb proper membrane function by applying similar mechanisms (3, 8–10). Importantly, membrane-active antibiotics even enable eradication of biofilms and other non-replicating cells and were further shown to synergistically enhance the activity of other antibiotics (11, 12). The lack of ATP upon disruption of membrane integrity and the inability of the bacteria to protect themselves from the antibiotic via target mutations further reduce the risk of antibiotic resistance development (13, 14). Despite initial concerns of adverse events due to missing selectivity for bacterial cells, membrane-active antibiotics are now considered as promising for the therapy of bacterial infections, including such being caused by antibiotic-resistant, biofilm-associated and non-replicating bacteria (15). According to recent data, the broad-spectrum atypical tetracycline chelocardin (CHD) and its derivative CDCHD (Fig. 1) have their target sites in the cell envelope, too (16, 17). CHD is a broad-spectrum antibiotic produced by the actinobacterium Amycolatopsis sulfurea and belongs to the class of the atypical tetracyclines (18, 19). CDCHD is a modified derivative of CHD with an extended activity spectrum due to its ability to evade antibiotic efflux (20, 21). Although atypical tetracyclines share the basic tetracycline scaffold, they do not primarily target the ribosomal 30S subunit as seen for typical tetracyclines (17, 22, 23). Instead, CHD was proposed to have a dose-dependent dual mode-of-action targeting the cell envelope first and, at higher concentrations, hampering protein biosynthesis (17). It is proposed that the antibiotic effect of the CHD derivative CDCHD solely relies on its effect on the bacterial cell envelope (16). In line with this, fluorescently-tagged CHD was found to accumulate in the membrane of Bacillus subtilis (17). However, the exact target of the CHDs remains unknown.

Due to the new target of CHDs, common tetracycline resistance markers, such as *tetM* and *tetW* protecting the ribosomes and occasionally found in many different bacteria, do not reduce the antimicrobial activity of CHDs. More importantly, common antibiotic efflux transporters, such as TetA, TetB, MexEF, and MeXY, similarly do not protect bacteria against CHDs, particularly CDCHD. For instance, CDCHD also inhibited the growth of strains with reduced sensitivity against CHD, e.g., a Klebsiella pneumoniae strain equipped with the AcrAB-TolC efflux (21). Until today, ChdR, an efflux system found in the genome of the producing strain Amycolatopsis sulfurea, is the only identified resistance mechanism against CHDs (21).

Here, we studied the response of the important anaerobic pathogen Clostridioides difficile to sublethal concentrations of CDCHD. C. difficile has become one of the most important nosocomial pathogens, and community-acquired forms of C. difficile infections (CDIs) have likewise become common (24). CDIs often come along with a significant health decline and high economic burden resulting from high hospitalization rates (25, 26). Consequently, there is an urgent need for improvements in infection management and treatment, which implies a more sophisticated understanding of the pathogen and its defense strategies. Due to their broad-spectrum activity, CHD and its derivatives are not suitable for the therapy of C. difficile infections. However, increased understanding of how C. difficile reacts to surface-active compounds is of great interest. The data presented in this study provide insights into the response of C. difficile to the surface-active antibiotic CDCHD and concomitant dissipation of the proton motive force.

## RESULTS AND DISCUSSION

### Anaerobe bacteria are susceptible to CDCHD and CHD.

CHDs have been shown to be active against several pathogenic Gram-positive and Gram-negative bacteria (21). However, susceptibility of anaerobic bacterial species devoid of a membrane-associated respiratory chain has not been shown yet. Therefore, we determined the MICs of CDCHD against some anaerobic representatives including five C. difficile isolates from human and porcine origin and five different ribotypes. MICs were similar to those observed for aerobic bacteria (21) and ranged from 2 to 4 μg/mL with the exception of Bifidobacterium longum, which was already inhibited at 0.5 μg/mL (Table S1 in the supplemental material). The five C. difficile strains were further tested for their susceptibility to the lead compound CHD, revealing similar MIC values for both compounds (Table S1).

10.1128/msphere.00302-22.3 TABLE S1 Minimal inhibitory concentrations of anaerobic bacteria to chelocardins. Minimal inhibitory concentrations [in μg/mL] were determined after 24 h of growth in an anaerobic environment using serial broth dilution assays. Na-CHD/citrate = chelocardin; Na-CDCHD = amidochelocardin. Download Table S1, XLSX file, 0.01 MB. Copyright © 2022 Brauer et al. 2022 Brauer et al. https://creativecommons.org/licenses/by/4.0/ This content is distributed under the terms of the Creative Commons Attribution 4.0 International license.

Since most anaerobes, such as clostridia, do not have a respiratory chain but alternative integral membrane components to build up their proton motive force and reveal different lipid membrane compositions (27, 28), the activity of CHDs against anaerobes is relevant to understand their membrane-directed antibiotic mechanism.

### The proteome response of C. difficile to CDCHD stress.

In the light of the promising role of membrane-active antibiotics and the potentially new mechanism of CDCHD, we analyzed how C. difficile strain 630 adapts its proteome in the presence of increasing concentrations of CDCHD. CDCHD was chosen due to its improved activity against antibiotic-resistant pathogens (21) and its single mode-of-action allowing the unbiased analysis of C. difficile*’s* response to membrane stress. Briefly, C. difficile 630 was grown to mid-exponential phase and stressed with three concentrations of CDCHD (0.75 μg/mL, 1.0 μg/mL and 1.5 μg/mL) (Fig. S1A in the supplemental material). Cells were harvested after 90 min, and the changes in the proteome of the pathogen following CDCHD stress were analyzed by mass spectrometry. In total, more than 1,800 proteins were identified (Fig. S1B). The most pronounced effects of CDCHD treatment were observed for the highest CDCHD concentration, respectively (Fig. S1C).

10.1128/msphere.00302-22.1 FIG S1 Proteomics analysis of C. difficile 630 to 0.75, 1.0 and 1.5 μg/mL amidochelocardin. (A) The stress response of C. difficile 630 to 0.75, 1.0 and 1.5 μg/mL amidochelocardin was analyzed by LC-MS/MS. Samples were taken 90 minutes following stress as indicated by the grey triangle. (B) Numbers of the identified proteins for the respective stress conditions are displayed as Venn diagram. (C) Proteins significantly higher abundant after treatment with 1.5 μg/mL *vs.* untreated controls are displayed as Volcano plot (log2-fold-change threshold = 1, adj. *p* value threshold = 0.05). Download FIG S1, TIF file, 1.3 MB. Copyright © 2022 Brauer et al. 2022 Brauer et al. https://creativecommons.org/licenses/by/4.0/ This content is distributed under the terms of the Creative Commons Attribution 4.0 International license.

**FIG 2:**
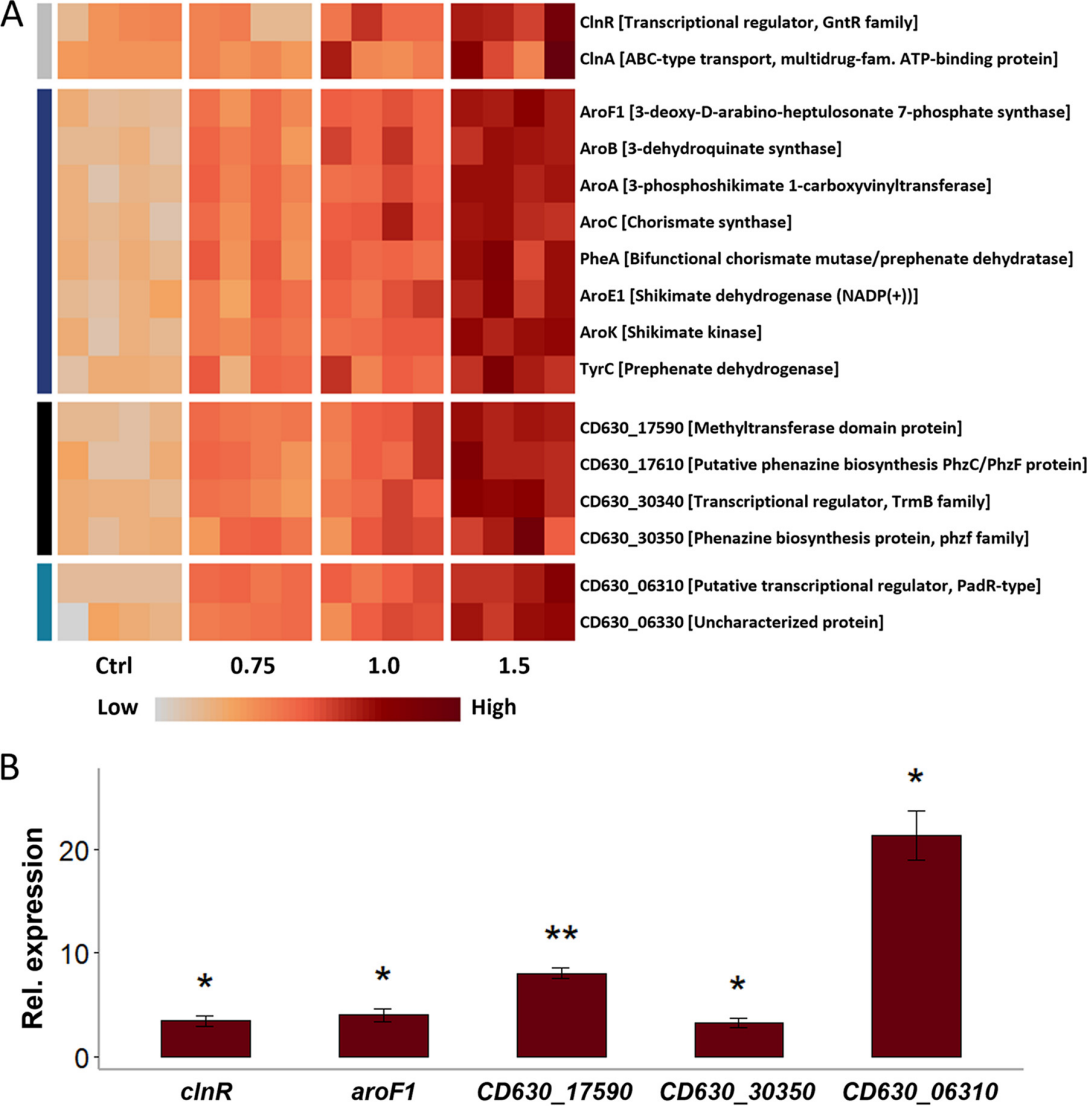
The proteome response of C. difficile to increasing concentrations of amidochelocardin. (A) C. difficile 630 was exposed to three concentrations of amidochelocardin (0.75, 1.0 and 1.5 μg/mL) and its proteome response to the antibiotic stress was analyzed by LC-MS/MS in comparison to non-treated cells (“Ctrl”). Proteins more abundant after stress with a log_2_ fold change ≥ 1 and a *P* value ≤ 0.05 were considered significantly enriched. They belong to four functional categories. Their abundance levels in the four independent biological replicates are displayed in a heatmap, where light colors represent lower abundance and dark red colors represent higher abundance. (B) The relative mRNA expression levels of selected genes in C. difficile 630 treated with 1.5 μg/mL amidochelocardin versus untreated cells were quantified by qPCR. Significant changes compared to control conditions are indicated by asterisks: *, *P* value ≤ 0.05; **, *P* value ≤ 0.01.

The stress response of C. difficile to CDCHD was characterized by a few distinct changes not observed in response to other antibiotics analyzed previously using similar approaches (29–31). First and most prominent, two proteins, which show amino acid sequence similarity to phenazine biosynthesis proteins from other bacterial species, CD630_17610 and CD630_30350, respectively, were significantly higher abundant in CDCHD-treated cells (log_2_ fold-change threshold ≥ 1, adj. *P* value threshold ≤ 0.05). Also additional proteins encoded nearby to each putative phenazine biosynthesis-like protein, CD630_17590 and CD630_30340, respectively, were higher abundant upon CDCHD stress (Fig. 2A, Table S2 in the supplemental material). Second, ClnA from the ClnAB antimicrobial peptide efflux system, previously described as specifically induced in response to antimicrobial peptides such as LL-37 (32), and the transcriptional regulator ClnR were found in higher amounts in CDCHD-treated cells (Fig. 2A, Table S2). Third, proteins from the operon encoding enzymes required for chorismate and aromatic amino acid biosynthesis were higher abundant after CDCHD treatment (Fig. 2A, Table S2). Fourth, a PadR-type transcriptional regulator, and a putative DNA alkylation repair protein encoded adjacent of the PadR-type regulator were found in elevated concentrations (Fig. 2A, Table S2). Finally, a leucine-sodium symporter was significantly lower abundant. Additionally, several membrane and membrane transport-associated proteins were identified in untreated cells but not in cells treated with the highest concentration of CDCHD (Tables S3 and S4). A complete overview of all identified proteins and their abundance in the different conditions is presented in the supplementary material (Table S4).

10.1128/msphere.00302-22.4 TABLE S2 Major findings from the proteome analysis of amidochelocardin stress in C. difficile. Proteins belonging to two predicted and three putative operons were found in significantly elevated amounts in amidochelocardin-treated C. difficile cultures compared to the untreated controls (log2-fold-change threshold ≥ 1, adj. *p* value threshold ≤ 0.05). Download Table S2, XLSX file, 0.01 MB. Copyright © 2022 Brauer et al. 2022 Brauer et al. https://creativecommons.org/licenses/by/4.0/ This content is distributed under the terms of the Creative Commons Attribution 4.0 International license.

10.1128/msphere.00302-22.5 TABLE S3 Proteins significantly less abundant or only identified in the controls. Proteins that could not be identified under at least one stress condition or were significantly lower abundant upon stress with at least one concentration of CDCHD including several transport-associated and membrane proteins. Download Table S3, XLSX file, 0.02 MB. Copyright © 2022 Brauer et al. 2022 Brauer et al. https://creativecommons.org/licenses/by/4.0/ This content is distributed under the terms of the Creative Commons Attribution 4.0 International license.

10.1128/msphere.00302-22.6 TABLE S4 Table of all proteins identified in the proteome experiments. Proteins were considered as identified if they were identified in at least three out of four biological replicates. Log2 fold changes were calculated for proteins if a protein was identified in both conditions to be compared. Proteins only identified in treated cells are listed as “ON” whereas proteins identified in untreated cells only are listed as “OFF”. Download Table S4, XLSX file, 0.5 MB. Copyright © 2022 Brauer et al. 2022 Brauer et al. https://creativecommons.org/licenses/by/4.0/ This content is distributed under the terms of the Creative Commons Attribution 4.0 International license.

The evenly enrichment of proteins from five putative transcriptional units led to speculate that the stress response of C. difficile 630 to CDCHD is regulated on transcriptional level. Therefore, mRNA levels of one gene of each putative operon were compared between untreated cells and cells exposed to 1.5 μg/mL CDCHD for 10 min. The obtained results validate that CDCHD induces the transcription of the five operons of interest (Fig. 2B, Table S5 in the supplemental material).

10.1128/msphere.00302-22.7 TABLE S5 qPCR data for five selected genes of interest. Expression of five selected genes and the house keeping gene *codY* was analyzed by qPCR in three biological replicates in three technical replicates. The relative expression of a gene was calculated according to the Pfaffl method, which is based on the average Ct values of the gene of interest and of the house keeping gene under control and stress conditions taking into the account the slope of the standard curve. Download Table S5, XLSX file, 0.04 MB. Copyright © 2022 Brauer et al. 2022 Brauer et al. https://creativecommons.org/licenses/by/4.0/ This content is distributed under the terms of the Creative Commons Attribution 4.0 International license.

Interestingly, these data are only to a small extent in line with previously published stress signatures of B. subtilis to CHD and CDCHD (16, 17). The effects listed above were not observed in B. subtilis whereas some of the effects observed in B. subtilis were not observed in C. difficile. This might be due to the different gene repertoire of the species and differences in the experimental setup. Stepanek et al. and Senges et al. used a pulse-chase 2D-gel-based approach and focused on proteins synthesized within the first 10 min following stress to study the compound’s mode-of-action (16, 17). Our data, on the other hand, were obtained by using a gel- and label-free LC-MS/MS approach that aimed to analyze how C. difficile has adapted its proteome after 90 min of growth in the presence of CDCHD.

However, all three CHD/CDCHD stress response signatures indicate impaired membrane integrity as evidenced, for example, by the increased production of ClnA and ClnR. The ClnAB efflux system was previously shown to selectively respond to cationic antimicrobial peptides (CAMPs) but not to other antimicrobials, like lysozyme, nisin and vancomycin (32). CAMPs attack bacteria using various mechanisms, including pore formation and membrane depolarization as well as membrane-independent mechanisms (7, 33). Surprisingly, ClnAB was specifically induced in C. difficile by LL-37, but provided only weak protection against CAMPs, whereas the global ClnR-mediated redirection of the cellular metabolism was suggested to support adaptation to the host environment (32). The presented data indicate that the specificity of ClnRAB to CAMP-stress needs to be revisited.

### CDCHD disrupts C. difficile*’s* membrane potential without affecting membrane barrier function.

**FIG 3:**
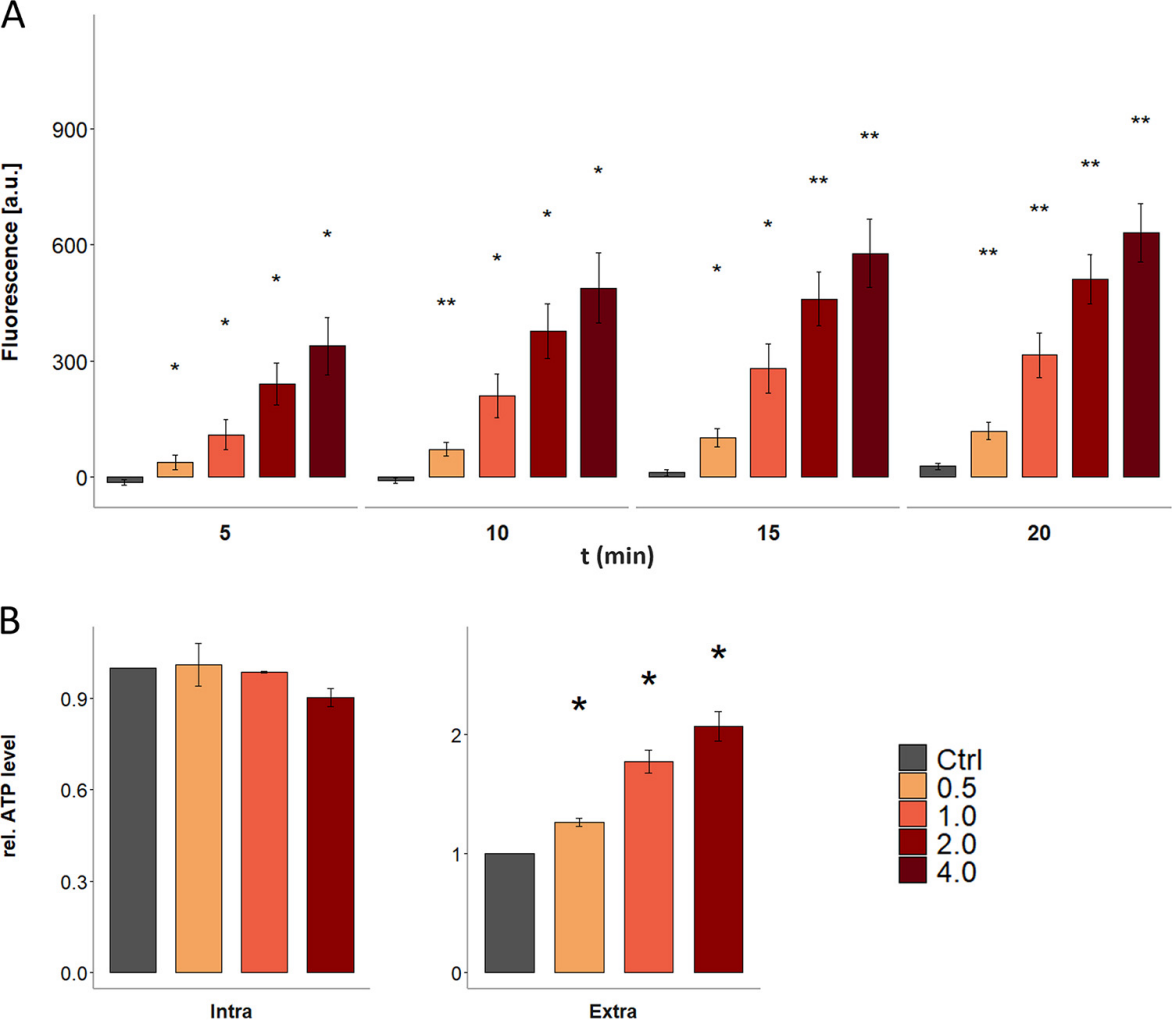
Dissipation of the proton motive force and ATP leakage in response to amidochelocardin. (A) Dissipation of C. difficile*’s* membrane potential after treatment with increasing concentrations of amidochelocardin (0.5, 1, 2 and 4 μg/mL) was monitored for 20 min in 5 min intervals using the fluorescent dye DISC_3_(5). Values are given in artificial units [a.u.] and represent means of three biological replicates. (B) Intra- and extracellular ATP levels were determined 90 min after treatment with increasing concentrations of amidochelocardin (0.5, 1, and 2 μg/mL) using the luminescence based CellTiter-Glo 2.0 Assay from Promega. Luminescence values are given in artificial units [a.u.] and represent means of three biological replicates. Significant changes compared to control conditions are indicated by asterisks. [a.u.] = artificial units; *, *P* value ≤ 0.05; **, *P* value ≤ 0.01.

Next, we determined the membrane potential of C. difficile as well as its intra- and extracellular ATP levels following CDCHD treatment to further characterize its antimicrobial effect. Together with the pH difference across the bacterial membrane (ΔpH), the membrane potential Δψ, referring to the distinct localization of ions, such as sodium and potassium, across the cytoplasmic membrane, forms the proton motive force, which is the central driver of ATP generation, membrane transport and flagellar activity in bacterial cells. The proton motive force can be quantified using the fluorescent dye DISC_3_(5), which accumulates inside polar membranes where its signal is partially quenched (34). Dissipation of the membrane potential Δψ results in release of the dye and a fluorescent signal while dissipation of ΔpH results in an increase of Δψ to counteract loss of the ΔpH and increases quenching of the DISC_3_(5) signal (34). Using DISC_3_(5), it could be observed that C. difficile*’s* membrane potential was indeed steadily dissipated with increasing concentrations of CDCHD, as reflected by the increase in the fluorescent signal (Fig. 3A). Only slightly increased ATP levels in the extracellular space observed in C. difficile cultures in the presence of CDCHD and no significant reduction of intracellular ATP levels further support previous findings that CDCHD kills via a pore-independent mechanism (Fig. 3B). In line with this, transmission electron microscopy (TEM) analysis of CDCHD-treated C. difficile cells further revealed that CDCHD did not substantially affect cell morphology (Fig. S2 in the supplemental material). Dissipation of the membrane potential might result from selective exchange of ions or distortion of membrane integrity, which has previously been observed for compounds that accumulate at the lipid water interface of the membrane, thereby hampering membrane fluidity, formation of functional membrane microdomains or affecting membrane thickness (35).

10.1128/msphere.00302-22.2 FIG S2 Transmission electron micrographs of C. difficile in the presence and absence of amidochelocardin. C. difficile was cultivated without (A) or in the presence of 1 μg/mL (B), 1.5 μg/mL (C) and 2 μg/mL (D) amidochelocardin for 90 minutes. None of the tested antibiotic concentrations had an effect on cell morphology. Scale bar: 500 nm. Download FIG S2, TIF file, 1.5 MB. Copyright © 2022 Brauer et al. 2022 Brauer et al. https://creativecommons.org/licenses/by/4.0/ This content is distributed under the terms of the Creative Commons Attribution 4.0 International license.

**FIG 4:**
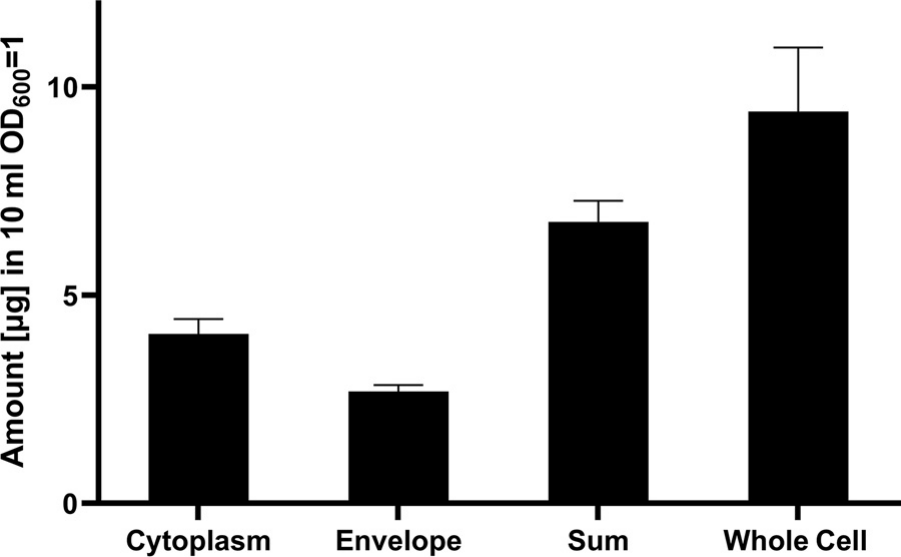
The proportion of amidochelocardin, which is retained within the cell envelope compared to the cytoplasm, was determined by LC-MS. For comparison, the summed up amounts of cytoplasm and envelope and the amount quantified in the “whole cell” fraction are displayed. Values present means of three biological replicates.

The enrichment of an antibiotic at a specific cellular compartment is another factor that determines efficacy. To probe the localization of CDCHD, a fractionation coupled to LC-MS/MS was applied to quantify its content in the cytoplasm and the cell envelope fraction of C. difficile following an incubation time of 10 min. The analysis revealed that a substantial amount of the compound was retained within the membrane (Fig. 4). Although the absolute amount of CDCHD was higher in the (much larger) cytoplasm, the cytoplasm-to-envelope ratio of 60:40 was substantially lower compared to other antibiotics like tetracycline (80:20) or erythromycin (99:1) that were previously studied in Escherichia coli (36).

### CDCHD treatment results in increased amounts of proteins required for production of aromatic compounds and increased intracellular tryptophan levels.

**FIG 5:**
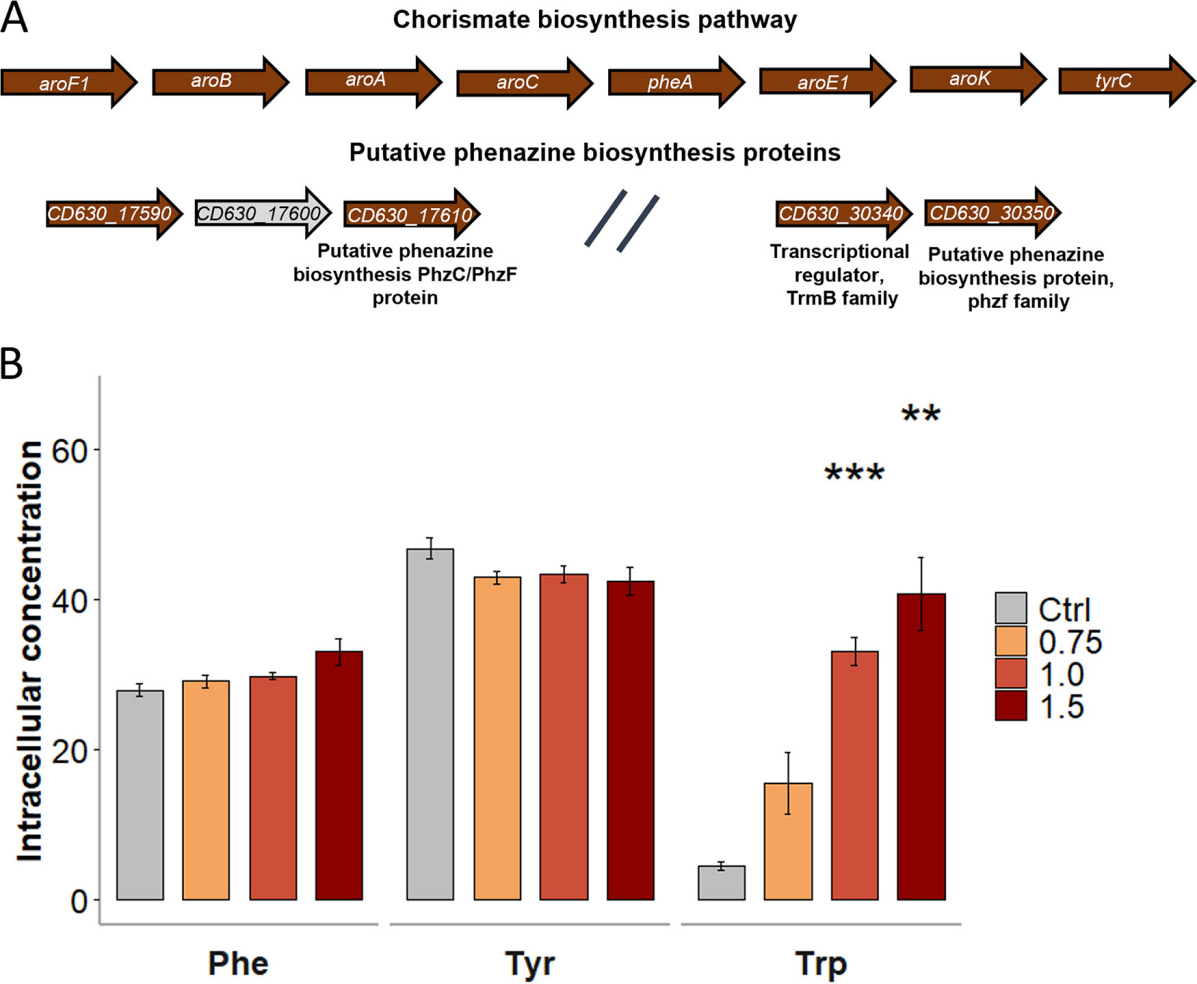
Increased abundance of proteins from the aromatic amino acid biosynthesis operon does not correlate with increased levels of phenylalanine and tyrosine. (A) In C. difficile the proteins for the synthesis of chorismate are encoded in one operon, which additionally comprises the genes encoding for phenylalanine and tyrosine biosynthesis enzymes PheA and TyrC. Two additional operons encode a putative phenazine biosynthesis protein each. In the presence of amidochelocardin, proteins from all three operons were found in significantly elevated amounts with the exception of the uncharacterized protein CD630_17600, which could not be identified. (B) Intracellular concentrations of the three aromatic amino acids were quantified in C. difficile in the presence of three different concentrations of amidochelocardin (0.75, 1.0 and 1.5 μg/mL) and in untreated cells. Significant changes are indicated by asterisks. *, *P* value ≤ 0.05; **, *P* value ≤ 0.01; ***, *P* value ≤ 0.001. Phe = phenylalanine, Tyr = tyrosine, Trp = tryptophan.

Finally, we addressed the question why C. difficile responds to dissipation of its membrane potential with increased production of proteins required for chorismate and aromatic amino acid biosynthesis as well as two putative phenazine biosynthesis proteins (Fig. 2; Fig. 5A). Phenazines, just as other aromatic compounds, such as ubiquinone and menaquinone as well as aromatic amino acid residues *per se*, are known to function as electron carriers, e.g., to shuttle electrons across the cell membrane (37–39). Phenazine production has been shown to be beneficial for bacteria, for instance, inside biofilms to shuttle electrons across the membrane when nutrients are depleted, and the membrane potential cannot be maintained (38). However, the production of phenazines has not been reported for C. difficile before this work. Most of these aromatic compounds as well as the two aromatic amino acids synthesized by C. difficile, tyrosine and phenylalanine, share chorismate as a common precursor. We speculated that the increased abundance of the chorismate biosynthesis proteins either provided chorismate for aromatic amino acid biosynthesis or alternatively for production of putative phenazine-like molecules. We therefore quantified relative concentrations of the aromatic amino acids, tyrosine, and phenylalanine, by a comparative GC-MS approach. The GC-MS data revealed that CDCHD treatment did not affect the relative intracellular tyrosine or phenylalanine levels (Fig. 5B). In contrast, the relative concentrations of tryptophan were significantly elevated upon CDCHD treatment (Fig. 5B). Interestingly, Saccharomyces cerevisiae similarly accumulates tryptophan in the cell within minutes upon SDS exposure conferring resistance to SDS via an unknown mechanism (40). Tryptophan is known for its important role in stabilizing membranes and anchoring of transmembrane proteins by being implicated in dipolar or hydrophobic interactions. Therefore, tryptophan is frequently incorporated in membrane proteins (41, 42). Its role in conferring resistance to membrane stress is, however, unclear. Likewise, the question whether C. difficile accumulates tryptophan via increased import or reduced utilization for protein synthesis cannot be assessed at this stage of the investigations. At least C. difficile 630 cannot synthesize tryptophan *de novo* due to the absence of the required genes, as reported before (43–45). The intermediates of the chorismate biosynthesis pathway could not be identified in the metabolite analyses. Likewise, a putative phenazine-like aromatic compound produced by C. difficile upon CDCHD treatment could not be identified by this initial analysis.

However, the finding that CD630_03610, a PadR-type domain transcriptional regulator, was also found in increased amounts upon CDCHD stress further supports the data discussed above. PadR-type regulators are involved in mitigating phenolic stress in other bacteria, and CD630_06310 might mediate mechanisms to protect C. difficile against the putative aromatic compound or CDCHD itself (46). However, the precise role of this regulator needs further investigation, as CD630_06310 is not homologous to the already characterized PadR regulator from C. difficile strain R20291 (47).

Membrane-active drugs, such as the non-steroidal anti-inflammatory drug carprofen and CHDs, are not only valuable due to their good bactericidal effect but also highly promising due to their synergistic effect with other antibiotics targeting macromolecule biosynthesis processes (11, 48, 49). For instance, a synergistic effect could be observed in Staphylococcus *pseudointermedius* upon co-treatment with carprofen, dissipating the membrane potential, and the tetracycline antibiotic doxycycline, inhibiting protein biosynthesis (48, 49). Finally, C. difficile was previously shown to be severely sensitive to membrane-active antibiotics pointing at the membrane as a very promising target in C. difficile (50).

In conclusion, our data provide valuable insights in how C. difficile potentially tolerates membrane-active compounds. The data set revealed that C. difficile responds to CDCHD-mediated dissipation of the membrane potential with increased production of proteins from the *clnRAB* operon, production of proteins involved in aromatic compound synthesis and accumulation of the aromatic amino acid tryptophan. Therefore, the data strongly suggest that aromatic compounds, such as tryptophan, and a putative phenazine-like molecule might play a role in mediating stress caused by dissipation of the membrane potential. Considering the membrane potential as effective antibiotic target, it might be worth to focus on the identification of membrane-located aromatic compounds in C. difficile and their role in the maintenance of the membrane potential. Additionally, it might be helpful to study the response of C. difficile to membrane-active antimicrobial peptides in more detail, as done for other bacterial pathogens (51–53).

## MATERIALS AND METHODS

### Preparation of natural products.

CHD and CDCHD were obtained by fermentation of the natural producer strain and a bioengineered strain of Amycolatopsis sulfurea, respectively, following procedures that are described elsewhere (20). Both derivatives were obtained as HCl salts in high purity (> 95%). These HCl salts were then converted into more stable and better water-soluble sodium salts. For this, a solution of 25 g/L sodium citrate dihydrate in double-distilled water was used to disperse CHD or CDCHD at a final *wt/wt* ratio of 1:1 (CHD-HCl/Na-citrate). The preparation was cooled to 4°C before adjusting pH 8.1–8.4 by the addition of 0.1 N NaOH. Powders of the following composition are obtained by lyophilization: Na-CHD/Na-citrate (1:1; *wt/wt*) and Na-CDCHD/Na-citrate (1:1; *wt/wt*).

### Bacterial strains and growth conditions.

Except for C. difficile strains 11S0047 and 12S0133, which were provided by Christian Seyboldt, Friedrich-Loeffler-Institute Jena, all strains were obtained from the DSMZ (German Collection of Microorganisms and Cell Cultures GmbH). All cultivation experiments were conducted in an anaerobic workstation (Whitley DG250 anaerobic workstation; 98% N_2_, 2% H_2_) at 37°C if not stated otherwise. C. difficile spores were inoculated in Brain Heart Infusion medium (Oxoid, Basingstoke, UK) with 0.1% taurocholic acid (Sigma-Aldrich, St. Louis, USA) to allow germination. All other bacteria were inoculated from frozen glycerol stocks.

### MICs.

MICs were determined in serial broth dilution assays. Briefly, Na-CHD/citrate, and Na-CDCHD/citrate were serially diluted in BHIS medium (BHI (Oxoid, Basingstoke, UK), 5% yeast extract, 1% l-cysteine, 0.1% vitamin K, 0.5% hemin(chloride); all from Carl Roth GmbH, Karlsruhe, Germany) in 96-well plates. Subsequently, plates were inoculated with 1:100 dilutions of overnight cultures of each individual strain and incubated at 37°C. MICs were determined after 24 h.

### Cultivation.

For all other experiments, C. difficile 630 was inoculated in a chemical defined medium, CDMM (54), to an optical density OD_600nm_ of 0.05. When approaching mid-exponential phase, cells were stressed with selected concentrations of CDCHD. Growth was either monitored until cells reached stationary phase or cells were processed for the respective experiments.

### Cell membrane permeability assay.

For cell membrane permeability assays cells were treated with 0.5 μM DISC_3_(5) solved in DMSO (Sigma-Aldrich, St. Louis, MO, USA). The dye was allowed to accumulate in the membrane for several minutes to allow for total quenching of the fluorescence signal. Subsequently, cells were treated with 0.5, 1, 2 or 4 μg/mL CDCHD or were left untreated and fluorescence was recorded with excitation at 500 nm and emission at 675 nm in a SynergyMx Microplate reader (BioTek Instruments, Winooski, VT, USA) for 30 min. Statistical testing was performed using the R package “rstatix” and using the adjusted t-testings (55).

### Intracellular and extracellular ATP concentrations.

Intracellular and extracellular ATP levels were determined with the CellTiter-Glo 2.0 Assay according to the manufacturer’s recommendations (Promega, Madison, WI, USA). Briefly, cells in mid-exponential growth phase were treated with 0.5, 1, or 2 μL CDCHD or were left untreated. Ninety minutes after stress, 1 mL samples from each culture were harvested by centrifugation at 9,000 × *g*; 100 μL of supernatant or 100 μL of pellets suspended in an equal volume of CDMM were mixed with 100 μL of reagent. Signals were allowed to stabilize and luminescence was recorded in a SynergyMx Microplate reader (BioTek Instruments, Winooski, VT, USA). Statistical testing was performed using the R package “rstatix” and using the adjusted t-testings (55).

### Proteomics experiments.

**(i) Harvest.** For proteomic analysis, C. difficile cultures were exposed to 0.75, 1, and 1.5 μg/mL Na-CDCHD/citrate and cultivated for further 90 min in the presence of the antibiotic. Subsequently, stressed and control cultures were harvested by centrifugation at 10,000 × *g* and 4°C for 5 min, cells were washed once with TE buffer (50 mM Tris, pH 7.5, 10 mM EDTA) and resulting cell pellets were stored at −70°C.

**(ii) Extraction of proteins.** Proteins were extracted from cell pellets as described previously (56). Briefly, cells were resuspended in 1 mL TE buffer and subjected to mechanical disruption with 500 μL glass beads (0.1 to 0.11 mm, Satorius Stedim Biotech, Göttingen, Germany) in a FastPrep-24 5G homogenizer (MP Biomedicals, Santa Ana, CA, USA) in three cycles at 6.5 m/s for 30 s. Glass beads and cell debris were removed by three centrifugation steps at 20,000 × *g* at 4°C. Protein extracts were stored at −70°C. Protein concentrations were determined using Roti-Nanoquant (Carl Roth GmbH, Karlsruhe, Germany) according to the manufacturer’s instructions.

**(iii) LC-MS/MS sample preparation.** For LC-MS/MS analysis 50 μg of protein sample were digested on S-trap micro columns (ProtiFi, Huntington, NY, USA) according to the manufacturer’s instructions. Briefly, protein samples were filled up with SDS lysis buffer to a final concentration of 5% SDS. Proteins were reduced with 10 mM DTT (Sigma-Aldrich, St. Louis, MO, USA), alkylated with 20 mM IAA (Sigma-Aldrich, St. Louis, MO, USA) and acidified with phosphoric acid (Carl Roth GmbH, Karlsruhe, Germany). Protein samples were diluted with 100 mM TEAB, 90% methanol in a ratio of 1:7 prior to transfer onto the S-trap micro columns. Columns were washed four times with 100 mM TEAB, 90% methanol. Subsequently, trypsin (Promega, Madison, WI, USA) was added in a ratio of 1:50 at 47°C for 3 h. Digested peptides were eluted from the columns in three steps: i) 50 mM TEAB, ii) 0.1% acetic acid, and iii) 60% acetonitrile, 0.1% acetic acid. Eluted peptides were dried by vacuum centrifugation. For peptide purification and fractionation digested peptides were suspended in 300 μL of 0.1 trifluoroacetic acid (TFA, Sigma-Aldrich, St. Louis, MO, USA) and loaded on self-packed and equilibrated C-18 columns (Reprosil Gold 300C18, 5 μm; Dr. Maisch GmbH, Ammerbruch-Entringen, Germany) as done previously (53). Columns were washed once with MS-pure water prior to stepwise elution with increasing concentrations of acetonitrile in 0.1% triethylamine (Carl Roth GmbH, Karlsruhe, Germany) comprising eight elution steps with concentrations ranging from 5% to 50% of acetonitrile. Finally, fractions 1 and 5, 2 and 6, 3 and 7, and 4 and 8 of each individual sample were pooled, dried by vacuum centrifugation and suspended in 0.1% acetic acid directly prior to LC-MS/MS analysis.

**(iv) LC-MS/MS analysis.** For LC-MS/MS analysis peptides were separated by liquid chromatography using an EASY nLC 1200 directly coupled to a Q Exactive HF Hybrid Quadrupole-Orbitrap mass spectrometer (Thermo Fisher Scientific, Waltham, MA, USA). Peptides loaded onto self-packed analytical columns with integrated emitter (100 μm × 20 cm) containing C_18_ reverse phase material (3 μm, Dr. Maisch, Ammerbruch-Entringen, Germany) and were eluted using a 85 min gradient from 5% to 50% of acetonitrile, 0.1% acetic acid at a constant flow rate of 300 nl/min. Full survey scans were performed with a resolution of 60,000 in the range of 333 – 1,650 *m/z*. MS/MS scans were performed for the 15 most abundant precursor ions per scan cycle excluding unassigned charge states and singly charged ions and dynamic exclusion enabled for 30 s. Internal lock mass calibration was applied (lock mass 445.12003).

**(v) LC-MS/MS data analysis.** For database search and label-free quantification (LFQ) the MaxQuant proteomics software package was used (57, 58) (version: 1.6.10.43). A protein sequence database for C. difficile 630 containing 3762 entries was obtained from Uniprot on March 15, 2021 (UP000001978). Common contaminants and reverse sequences were added by the MaxQuant software. Parameters were set as follows: Trypsin cleavage with a maximum of two missed cleavages was assumed. Oxidation of methionine was set as variable modification and carbamidomethylation of cysteine was set as fixed modification. Default parameters were used for protein identification. For label-free protein quantification unique and razor peptides were considered with a minimum ratio count of 2. Match between runs was enabled with default settings within each sample group. C. difficile proteins were considered as identified if they were identified with at least two unique peptides in at least three out of four biological replicates. Averaged LFQ intensities were used to calculate log_2_ fold changes. For identification of significantly changed protein intensities the R package DEqMS was used (59). Most interesting proteins were depicted as heatmap using the R package “pheatmap” (60).

### qPCR analysis of selected genes.

Five genes from each of the five predicted/putative operons of interest were chosen for mRNA expression analysis via qPCR. Cells were cultivated as done for the proteomics experiment with the exception that cells were exposed for 10 min to 1.5 μg/mL CDCHD only. Treated as well as untreated cells were immediately cooled in liquid nitrogen and collected by centrifugation at 10,000 × *g* and 4°C for 3 min. RNA was extracted using TRIzol reagent (Invitrogen, Waltham, MA, USA) according to the manufacturer’s recommendations, solubilized in diethyl pyrocarbonate (DEPC)-treated water and subjected to DNase (Roche, Basel, Switzerland) treatment. The RevertAid RT kit (Thermo Fisher Scientific Baltics, Vilnius, Lithuania) was used for cDNA synthesis from 500 ng RNA. Quantitative PCR was performed with a qTOWER 3 quantitative PCR thermocycler (Analytik Jena, Jena, Germany) using the Luna Universal qPCR Master Mix (NEB GmbH, Frankfurt, Germany). Expression of *codY* was used as internal reference and relative expression was calculated according to Pfaffl (61). Statistical testing was performed using the R package “rstatix” applying adjusted t-testings (55).

### Targeted metabolomics analysis of aromatic amino acids.

**(i) Extraction of metabolites.** To quantify intracellular levels of aromatic amino acids and intermediates of the chorismate biosynthesis pathway, cells were treated as done for the proteomics approach (see Proteomics: Harvest). Cells were immediately cooled in liquid nitrogen, were collected by centrifugation and again cooled in liquid nitrogen. Subsequently, samples were prepared for and analyzed by GC-MS as done previously (62). Briefly, intracellular metabolites were extracted by three rounds of bead beating suspended in 800 μL of 60% ice-cold ethanol, 200 μL of an internal GC-MS standard (GC4; 20 nM (each) *N*,*N-*dimethyl-phenylalanine, *p*-chloro-phenylalanine hydroxide, norvaline, ribitol; Sigma-Aldrich, St. Louis, USA) and 500 μL of glass beads. 500 μL of CDMM or 500 μL of MS-pure water were included as medium or empty control. The cytosolic fraction was separated from the cell debris by centrifugation at 20,000 × *g* at 4°C for 5 min. Glass beads and cell debris were washed with 800 μL of MS-pure water by an additional round of bead beating followed by centrifugation at 20,000 × *g* at 4°C for 5 min. Supernatants of both rounds were pooled and 5 mL of MS-pure water were added to every sample. Samples were frozen at −80°C and completely frozen samples were lyophilized in a freeze dryer (Alpha 1–2 LDplus, Christ, Osterode am Harz, Germany) overnight. Dried cytosolic extracts were suspended in 500 μL of MS-pure water and transferred to 1.7 mL low binding tubes. Samples were allowed to freeze again at −80°C before they were again lyophilized.

**(ii) GC-MS analysis.** Samples were analyzed by GC-MS as described by Liebeke et al. (63). Lyophilized samples were derivatized with 40 μL methoxyaminehydrochloride for 90 min at 37°C, mixed with 80 μL N-methyl-N-trimethylsilyltrifluoroacetamide and incubated for 30 min at 37°C. Subsequently, 2 μL of each sample were injected into an Agilent 6890N GC system with SSl-injector [Split 1:25 at 250°C; inlet split flow: 20 mL/min; carrier gas: helium 1 mL/min (60 kPa) at 110°C; pressure rise: 6 kPa/min] coupled to an Agilent 5973 Network MSD mass selective detector (Agilent Technologies, Santa Clara, CA, USA) operated in electron ionization mode with an ionization energy of 70 eV. Chromatographic separation was achieved using a 30-m DB-5MS column (30 m x 0,25 mm x 0,25 μm; Agilent Technologies, Santa Clara, CA, USA) using an oven program comprising the following steps: i) an initial temperature hold at 70°C for 1 min, ii) stepwise heating with 1.5°C/min up to 76°C stepwise heating with 5°C/min up to 220°C, iii) stepwise heating with 20°C/min up to 320°C, iv) a hold at 320°C for 5 min. Finally, analytes were transferred to the mass selective detector via the transfer line at 280°C and full scans were performed from 50 to 550 *m/z* at a scan rate of 2.74 scans per second with a 6 min solvent delay.

**(iii) GC-MS data analysis.** Raw GC-MS data were processed with MassHunter version B 8.00 software (Agilent Technologies, Santa Clara, CA, USA). Retention times and fragmentation patterns of detected metabolites were first aligned to retention times and fragmentation patterns of internal standards and searched against the NIST 2017 mass spectral database 2.0 d (National Institute of Standards and Technology, Gaithersburg, TN, USA) (64). Finally, relative concentrations of identified metabolites were calculated based on peak areas of the quantifier ion of each metabolite normalized to the peak areas of the quantifier ion of the internal standard using MassHunter software. Statistical testing was performed using the R package “rstatix” and using the adjusted t-testings (55).

### Localization of CDCHD.

**(i) Harvest.** To quantify the amount of CDCHD retained in the cytoplasmic membrane, cells were grown to an OD_600nm_ of 0.8 and were treated with 10 μg/mL CDCHD for 10 min. Subsequently, samples were fractionated and analyzed by modifying a previously established protocol (36). Treated and untreated cells were collected by centrifugation for 5 min at 10,000 × *g* and 4°C. Cell pellets were suspended in 2 mL TBS (50 mM Tris, pH 7.6, 150 mM NaCl), were split into two equal subsamples and were centrifuged again for 5 min at 4,500 *g* and 4°C. Cells were washed once with 1 mL 25 mM Tris, pH 7.4 followed by centrifugation for 5 min at 4,500 *g* and 4°C. Supernatants were discarded.

**(ii) Cellular fractionation.** For cell disruption, cells were subjected to ultrasonication in 190 μL 10 mM Tris, pH 7.4 in five cycles à 30 s at an amplitude of 60% with pulse ratio 0.1/0.5 s (Sonopuls Ultrasonic Homogenizer, Bandelin, electronic GmbH & Co. KG, Berlin, Germany). To digest DNA in the samples, samples were incubated with 2.8 ng/mL DNase I (Roche, Basel, Switzerland) for 15 min at 37°C and 1,000 rpm. Following DNase treatment, one subsample of each sample was directly stored at −20°C (“whole cell”). The remaining samples were transferred to ultracentrifuge tubes and cell envelope and cytoplasmic fractions were separated by centrifugation for 1 h at 100,000 × *g* and 4°C (Sorvall Discovery M150 SE, Thermo Fisher Scientific, Waltham, MA, USA). Subsequently, the supernatants were retained as cytoplasmic fraction (“cytoplasmic”). Pellets were washed once by carefully applying 200 μL of 10 mM Tris, pH 7.4 and centrifugation for 1 min at 16,000 *g* and 4°C. Supernatants were discarded and pellets were suspended in 200 μL 0.5 mM MgSO_4_ in an ultrasonic bath (“pellet”).

**(iii) LC-MS/MS analysis.** For quantification of CDCHD uptake, liquid chromatography (Agilent 1290 Infinity II, Technologies, Santa Clara, CA, USA) coupled to linear ion trap quadrupole mass spectromety (Absciex QTrap6500, Darmstadt, Germany) was used. First a protein precipitation step was performed using 80 μL of sample mixed with 80 μL H_2_O, 120 μL of ACN and 120 μL of MeOH. Subsequently the mixture was centrifuged at 2250 × *g* for 60 min at 4°C. Next, 320 μL of supernatant were dried overnight in a CentriVap equipped with a −50°C cold trap (Labconco, Kansas, MO, USA). Prior to LC injection samples were reconstituted in 40 μL MS-Buffer (40% H_2_O, 30% ACN and 30% MeOH), containing 100 ng/mL Glipizide as internal standard (IS). LC separation was achieved using a reversed phase column (Phenomenex Gemini 3 μm NX-C18 110A; 50 × 2 mm) equipped with a respective guard column (5 × 2 mm) (Phenomenex, Torrance, CA, USA) at a flow rate of 700 μL/min and a linear gradient starting at 1 min 5% B, up to 5 min 95% B and additional 1 min 95% B (A: H_2_O + 0.1% HCOOH; B: ACN + 0.1% HCOOH). Targeted analyses in negative ion mode were done using multiple reaction monitoring. The following MRM settings were used for detection: IS was measured as *m/z*: 443.9, with fragments: *m/z*: 319.1 (Declustering Potential −66; Colision Energy: −26; Cell Exit Potential: −21) and *m/z*: 170.1 (Declustering Potential -66; Colision Energy: −40; Cell Exit Potential: −7). For CDCHD (*m/z*: 411.1), fragments *m/z*: 269.1 (Declustering Potential -5.0; Colision Energy: −20; Cell Exit Potential: −13) and *m/z*: 141.0 (Declustering Potential −5.0; Colision Energy: −20; Cell Exit Potential: −9) were quantified using the software Analyst 1.6.3 and Multiquant 3.0 (AB Sciex Germany GmbH, Darmstadt, Germany).

### TEM.

Cells were treated as done for the proteomics experiment and were grown in the presence of the antibiotic for 90 min (see Proteomics: Harvest). Subsequently, stressed and unstressed cells were harvested by centrifugation at 2,250 × *g* and 4°C for 10 min, washed once with PBS and harvested again by centrifugation at 3,500 × *g* and 4°C for 5 min. Pellets were suspended in 200 μL PBS and 1 mL fixative (100 mM cacodylate buffer, 2% glutaraldehyde, 2% paraformaldehyde, 5 mM calcium chloride, 10 mM magnesium chloride, 50 mM sodium azide; pH 7.4) was added. Cells were incubated for 10 min at 20°C and shifted to 4°C overnight with slow agitation. Next day, samples were centrifuged at 6,000 × *g* and 4°C for 3 min, washed three times with washing buffer (100 mM cacodylate buffer; pH 7.4) for 3 min each time, and after a final centrifugation step at 6,000 × *g* and 4°C for 3 min embedded in low gelling agarose. Samples were proceeded further according to Metzendorf et al. (65) and analyzed with a transmission electron microscope LEO 906 (Carl Zeiss Microscopy GmbH, Oberkochen, Germany). For acquisition of the images, a wide-angle dual speed CCD camera Sharpeye (Tröndle, Moorenweis, Germany) was used, operated by the ImageSP software. All micrographs were edited by using Adobe Photoshop CS6.

### Statistical analyses and visualization.

If not stated otherwise, statistical analyses were performed using R package “rstatix” (55) and data were visualized using the R package “ggplot2,” “ggpubr” and “RColorBrewer” (66, 67).

### Data availability statement.

The mass spectrometry proteomics data have been deposited to the ProteomeXchange Consortium via the PRIDE partner repository with the data set identifier PXD029250.
