# Supplementary figures and images for: Clostridioides difficile Modifies its Aromatic Compound Metabolism in Response to Amidochelocardin-Induced Membrane Stress
Source: mSphere. 2022 Aug 22;7(5):e00302-22. doi: 10.1128/msphere.00302-22 (PMC9599328; doi:10.1128/msphere.00302-22)

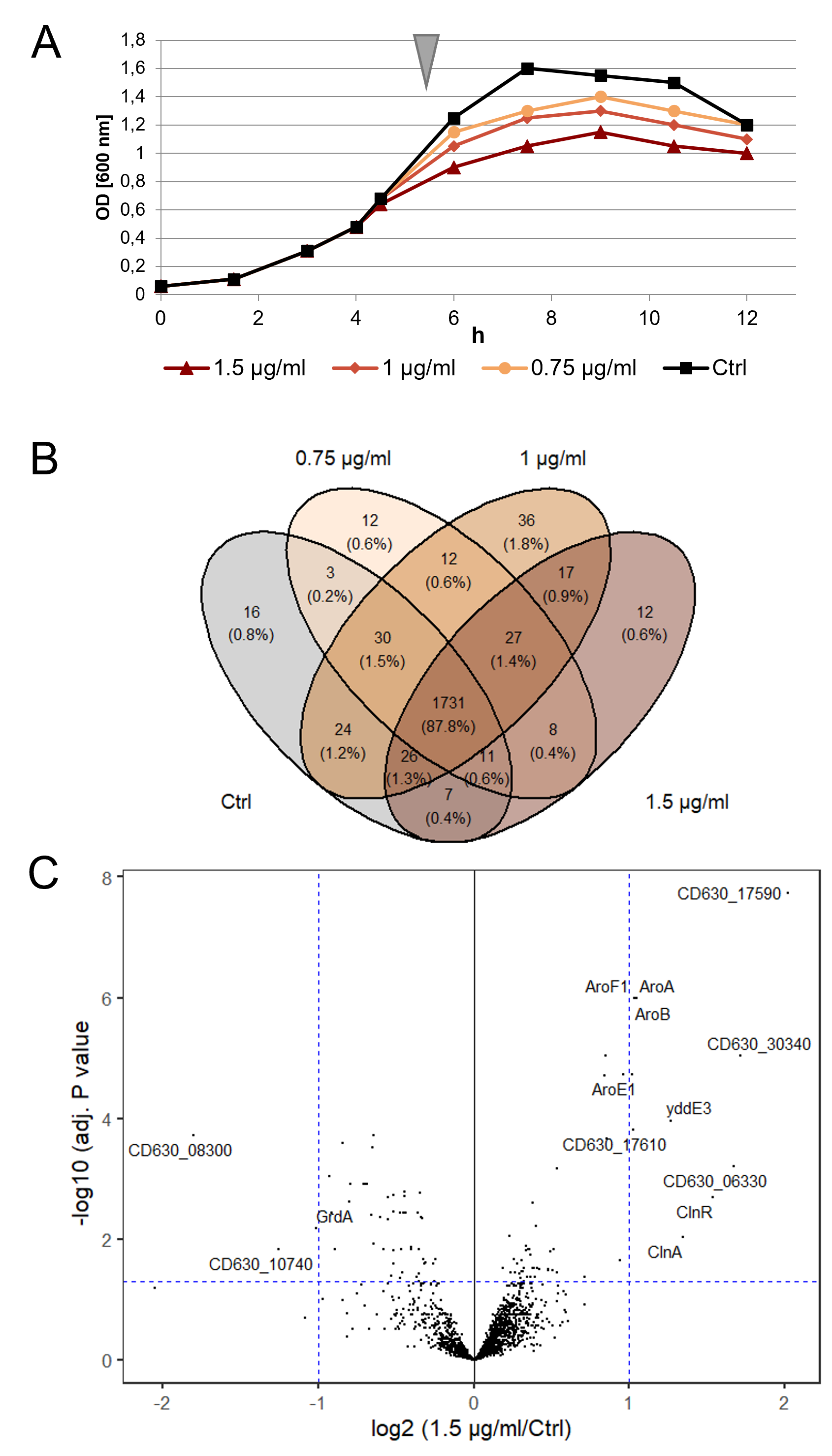

Supplement: FIG S1 [file msphere.00302-22-s0007.tif]

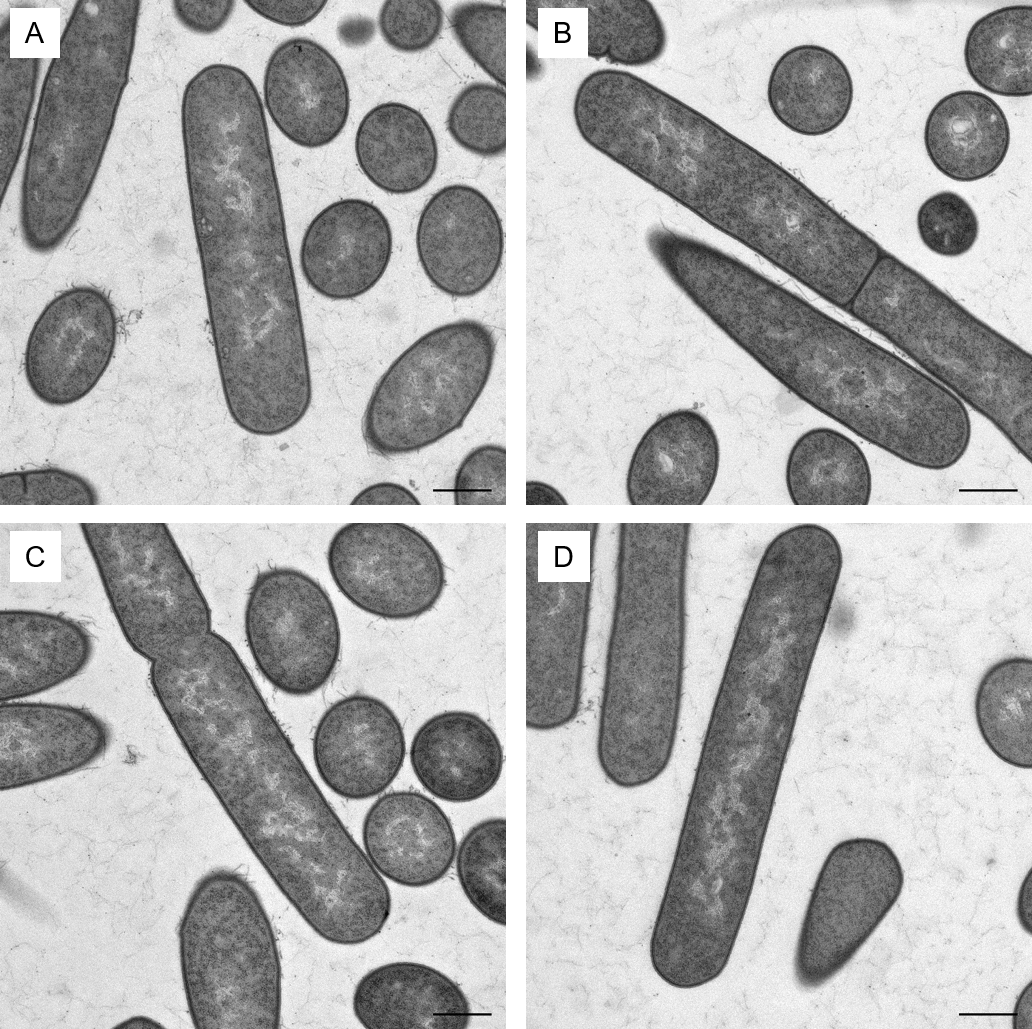

Supplement: FIG S2 [file msphere.00302-22-s0006.tif]
